# How to Group Genes according to Expression Profiles?

**DOI:** 10.1155/2011/261975

**Published:** 2011-12-20

**Authors:** Julio A. Di Rienzo, Silvia G. Valdano, Paula Fernández

**Affiliations:** ^1^Estadistica y Biometría, Universidad Nacional de Córdoba, 5000 Córdoba, Argentina; ^2^Departamento de Ciencias Naturales, Universidad Nacional de Río Cuarto, 5800 Río Cuarto, Argentina; ^3^Instituto de Biotecnología, INTA-Castelar, 1712 Castelar, Argentina

## Abstract

The most commonly applied strategies for identifying genes with a common response profile are based on clustering algorithms. These methods have no explicit rules to define the appropriate number of groups of genes. Usually the number of clusters is decided on heuristic criteria or through the application of different methods proposed to assess the number of clusters in a data set. The purpose of this paper is to compare the performance of seven of these techniques, including traditional ones, and some recently proposed. All of them produce underestimations of the true number of clusters. However, within this limitation, the *gDGC* algorithm appears to be the best. It is the only one that explicitly states a rule for cutting a dendrogram on the basis of a testing hypothesis framework, allowing the user to calibrate the sensitivity, adjusting the significance level.

## 1. Introduction

One of the main purposes of microarray experiments is to discover genes having differential expression level among a set a treatment conditions. Once the set of “candidates” genes is obtained, the problem of identifying those having a common response profile across experimental conditions remains open [1–3]. There are many strategies to proceed with. One of them is the exploration of gene's ontology; the others—and more commonly applied—are based on unsupervised classification algorithms (cluster analysis). The main purpose of clustering techniques is to arrange a number of instances to produce meaningful grouping of them. Hierarchical clustering methods not only allow to group genes but also to trace their relationships. The outcome of hierarchical methods is displayed as a binary tree called dendrogram. A key point for interpreting a dendrogram is to decide where to cut it. This decision is equivalent to determine the number of clusters in the dataset. The problem is to realize which instances belong to different groups and which seem to be different just as the result of sampling errors.

Several general-purpose methods have been proposed to estimate the optimal number of clusters in a dataset. The most popular are those introduced by Calinski and Harabasz [4], Hartigan [5], Sarle [6], and Kaufman and Rousseeuw [7].

Tibshirani et al. [8] proposed the *Gap* statistic as a method for assessing the number of clusters in a dataset. It compares the log of the within-cluster sum of squares against its expected value under a suitable null distribution. Authors exemplified its application to the discovery of groups in a hierarchical clustering of genes of a microarray experiment.

Another method, developed in the framework of large-scale gene-expression studies, is the algorithm called Hierarchical Ordered Partitioning and Collapsing Hybrid (*HOPACH*) [2]. It is a hierarchical tree of clusters and was developed for the purpose of discovering patterns within a hierarchical structure.

A different approach to the problem of grouping is the visualization of data as a sample of a mixture of populations. The classification is the result of a mixture model estimation and selection that takes into account not only different number of populations present in the sample and their location parameters but also the dispersion and correlation parameters. A procedure that is representative of this kind of methods is the algorithm *MClust* of Fraley and Raftery [9, 10], which is based on the modeling of a multivariate normal mixture.

Every method mentioned previously assumes that each instance is represented by a *p*-variate vector of attributes. In microarray experiments genes represent the instances and their expressions, observed in the contrasting experimental conditions, the *p*-variate vector of attributes. In this type of experiments there are, usually, several biological replicates for each experimental condition. None of the methods mentioned above makes an explicit use of those replicates. Valdano and Di Rienzo [11] proposed a multivariate generalization (*gDGC*) of a univariate pairwise comparison procedure [12] which uses replicates to estimate the cutting point of a dendrogram generated by a given linkage algorithm. In this way the procedure generates a partition within a hierarchical structure which is a nice property in the framework of microarray experiments data analysis.

 Considering the revisions of Tibshirani et al. [8], Lee et al. [1], Pollard and van der Laan [2], and Gentleman and Carey [3], regarding the problem of assessing the number of clusters in a dataset, we focused on the comparison of the last four methods mentioned before for estimating the number of clusters in a gene-expression matrix. However, we included general-purpose methods as reference. The comparison was done under a set of scenarios described in following sections.

## 2. Methods

Let the dataset {*x*_*ij*_}, *i* = 1,…, *n*, *j* = 1,…, *p* consist of *p* features measured on *n*-independent observations; that is, **X** is a *nxp* data matrix. Suppose that we have grouped the data into *k* clusters. Let **Z** be a cluster indicator matrix (*z*_*ir*_ = 1 if the *i*th observation belongs to the *r*th cluster, else *z*_*ir*_ = 0, *i* = 1,…, *n*; *r* = 1,…, *k*) and **C** a *k* × *p* matrix of cluster means:

(1) $$C={\left(Z^{\prime }Z\right)}^{-1}Z^{\prime }X.$$

Then, the pooled within-cluster sum of squares matrix is

(2) $$W={\left(X-ZC\right)}^{\prime }\left(X-ZC\right),$$

and the between-cluster sum of squares matrix is

(3) $$B=C^{\prime }Z^{\prime }ZC.$$

The within-cluster and the between-cluster sums of squares pooled over variables, for a given number *k* of clusters, are, respectively,

(4) W(k)=trace(W),  B(k)=trace(B).

The following methods, proposed to estimate the optimum number of clusters in a dataset, are compared. They are identified by the name of the algorithm which implements them or by the initials of their authors.

CH. Calinski and Harabasz [4] based the selection of the number of clusters on the maximization of the between/within-cluster sums of squares ratio. The criterion to choose *k* is the one that maximizes CH(*k*):

(5) $$\text{CH}\left(k\right)=\frac{B\left(k\right)/\left(k-1\right)\;}{W\left(k\right)/\left(n-k\right)\;}.$$

H. Hartigan [5] used the ratio between the within-cluster sums of squares of *k* and (*k* + 1) clusters suggesting the selection of *k* ≥ 1 as the minimum *k* for which the ratio is lesser than 10:

(6) $$\text{H}\left(k\right)=\frac{\left[\left(W\left(k\right)/W\left(k+1\right)\right)-1\right]}{\left(n-k-1\right)}.$$

CCC. Sarle [6] introduced the cubic clustering criterion based on the scaled *log*[1 − *E*(*R*2)/(1 − *R*2)], where *R* ^2^ is the proportion of variance accounted by the clusters and *E*(*R* ^2^) is the expected value of observed *R* ^2^ assuming that the data are uniformly distributed on a hypercube:

(7) $$\text{CCC}=\log\left[\frac{1-E\left(R^{2}\right)}{1-R^{2}}\right]\cdot \frac{\sqrt{np^{*}/2}}{{\left[(0.001+E\left(R^{2}\right)\right]}^{1.2}}.$$

p* is the dimensionality of the between-cluster variation. The criterion to choose the optimum number of clusters is to select the number that maximizes CCC. Maximum value of the CCC index lesser than 2 indicates that there is no evidence of the existence of clusters in the dataset.

CCCm. We also included a modified version of *CCC* for which the expected value of *R* ^2^ is calculated from the null distribution described as a uniform distribution over a box aligned with the principal components of the data as was proposed by Tibshirani et al. [8] for the *Gap* statistic.

Silh. Kaufman and Rousseeuw [7] introduced the *Silhouette* statistic, a measurement, calculated for each observation, based on a standardized difference between the average distance of the *i*th observation to each other in the same cluster *a*(*i*) and the average distance to the observations in the nearest cluster *b*(*i*):

(8) $$s\left(i\right)=\frac{b\left(i\right)-a\left(i\right)}{\max\left(a\left(i\right),b\left(i\right)\right)}.$$

They proposed to choose the optimal number of clusters as the value maximizing the average *silhouette*. It is implemented in the function *silcheck *in the *hopach R*-package [13].

Gap. Tibshirani et al. [8] used the *gap* statistic for estimating the number of clusters in a dataset. The *gap* statistic compares the log of the within-cluster sum of squares against its expected value under a suitable null distribution of the dataset:

(9) gap⁡(k)=E[log⁡(W(k))]−log⁡(W(k)).

The criterion to select the number *k* of clusters is, in this case, the lesser *k* such that gap⁡(*k*) ≥ gap⁡(*k* + 1) − *s*(*k* + 1), where *s*(*k* + 1) is the standard deviation of a prediction of *gap* when the number of clusters is (*k* + 1). For the calculation of *E*[log⁡(*W*(*k*))], Tibshirani et al. [8] generate a null distribution from a uniform distribution over a box aligned with the principal components of the data. The authors argued that this way of generating the data takes into account the shape of the distribution of the original observations and makes the procedure rotationally invariant, as long as the clustering method itself is invariant.

HOPACH. Pollard and van der Laan [2] proposed another application of the *Silhouette* statistics: the Hierarchical Ordered Partitioning and Collapsing Hybrid (HOPACH) procedure which iteratively applies a partitioning algorithm to produce a hierarchical tree of clusters. It is implemented in the *hopach* function of the *hopach R*-package [13]. At each node, a cluster is partitioned into two or more smaller clusters and, before the next partitioning step, any similar clusters are merged. The algorithm estimates the optimal number of clusters based on median split silhouette criterion. The function can be called passing to it a data frame or a distance matrix. We try both: passing the data frame of the average gene-expressions (*HOPACHc*) and the Mahalanobis distances matrix (*HOPACHm*). All other arguments were left to their default settings.

MClust. Fraley and Raftery [9, 10] proposed a method of clustering that is based on the assumption that the dataset is a sample of a multivariate normal mixture. The method fits a number of models for a number of populations differing not only in the location parameter but also in the variance-covariance matrix within a set of plausible simplified correlation structure. The model selection rule is based on the Bayesian Information Criterion. As part of the output of this method, the estimated number of clusters in the data is obtained. The input to this method is the matrix of average expression level for each gene (rows) on the different experimental conditions (columns). The routine is already implemented in *R* (*mclust R*-package). Within our simulation, it was called without any additional arguments except the data frame.

gDGC. Valdano and Di Rienzo [11] calculated a cutting point for a dendrogram, generated by a given linkage algorithm, based on the null distribution of the root node of the binary tree produced by the clustering procedure. The node in which two mean vectors—or a cluster of them—join have an associated measure that corresponds to the distance—calculated according to linkage algorithm—between the mean vectors or the clusters that the node is joining. The node in which all mean vectors join, to form a unique cluster, is the* root node*. In the UPGMA algorithm, if *S*_*M*_ and *S*_*L*_ are two different clusters, the distance between them is defined as follow:

(10) $$q=q\left(S_{M},S_{L}\right)=\frac{1}{\#\left(S_{M}\right)\#\left(S_{L}\right)}\overset{}{\underset{\begin{array}{c} {\overset{̅}{y}}_{i}\in S_{M}\\ {\overset{̅}{y}}_{j}\in S_{L} \end{array}}{\sum }}D_{ij},$$

where *D*_*ij*_ is the square root of Mahalanobis distance. If *S*_*M*_ and *S*_*L*_ are coincident, then *q*(*S*_*M*_, *S*_*M*_) = 0.

**Figure 1:**
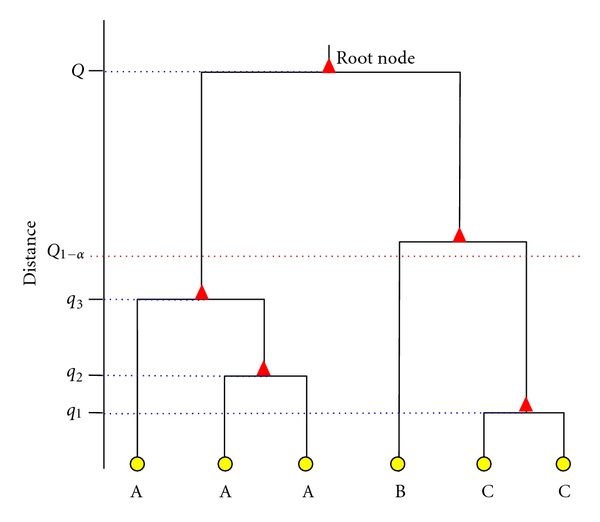
Dendrogram showing the relationships among mean vectors. Cut-off criterion obtained with the *gDGC* test—*Q*_1−*α*_—is indicated with a dotted line. At the bottom of the figure, different letters identify groups statistically differing in the population centroids at a significance level *α*.

The smallest value of *D*_*ij*_ will correspond to the pair of most similar mean vectors and the node that is formed will be at a distance *q*_1_ from the origin. The following distance—*q*_2_—is associated with the next node, which can join two different mean vectors or the cluster previously formed and another mean vector. At the end of the clustering algorithm, the last union will be at distance *q*_*k*−1_ and will be referred to as the distance to the root node (Figure 1). This distance can be seen as a realization of a random variable *Q*. The (1−*α*)-quantile of its distribution under the null hypothesis of equal population mean vectors can be used to construct a test of size *α*. Given *Q*_1−*α*_, as the *α*-level critical value, all *Q* ≥ *Q*_1−*α*_ will lead to the rejection of the null hypothesis. An *R* routine that calculates critical points of the null distribution of Q is freely available to download at: http://agro.uncor.edu/~estad/gDGCQ.r. A friendly implementation of *gDGC* for its application on a gene-expression matrix can be found in the free-software fgStatistics http://sites.google.com/site/fgstatistics/.

## 3. Simulated Data

The primary output of a microarray experiment is the gene-expression matrix (*GEM*). It is composed by *G* rows and *H* columns. *G* is the number of “genes” evaluated and *H* is the number of microarrays (treatments × replicates) used in the experiment. Usually *G* is bigger than *H* and varies between hundreds to tens of thousands. Candidate genes are those genes that are differentially expressed among “treatments”. The set of candidate genes is smaller than original set of genes and its size is around tens to hundreds of genes. This drastic reduction in the number of genes relays in the assumption that most of them remain unchanged under the experimental conditions contrasted.

To simulate the candidate genes expression matrices we considered two scenarios regarding the number of differentially expressed genes (100 and 300 genes), two levels for the number of clusters which have similar profile among treatments: 2 and 10, two levels for the number of treatment conditions: 3 and 5, and two levels for the number of replicates: 3 and 6. Anumber of genes, clusters, treatments, and replicates do not intend to cover all possibilities but common cases in microarray experiments. According to the number of differentially expressed genes (2), the number of clusters (2), the number of treatments (2), and then number of replicates (2), 16 scenarios were considered, For each scenario 10 simulated candidate-gene-expression matrixes (sGEM) were randomly generated.

Each sGEM was generated from the GEM of a self-self cDNA-microarray experiment dataset [14] according to Algorithm 1 described in the appendix. The algorithm relays on the availability of a residual gene-expression matrix (rGEM). This residual matrix was obtained from the GEM of the self-self experiment (s-sGEM) by centering by rows and columns and adding to each entry the mean of all entries. This way of obtaining an rGEM assumes that each entry (*Y*_*ij*_) in s-sGEM can be modeled as *Y*_*ij*_ = *μ* + *g*_*i*_ + *m*_*j*_ + *ε*_*ik*_, *i* = 1 … *G*, *j* = 1 … *H*, where *μ* is a common mean, *g*_*i*_ is the effect of the *i*th gene, *m*_*j*_ is the *j*th microarray's effect, and *ε*_*ij*_ is a random error with zero mean. The resulting rGEM was a 3830 (rows) by 10 (columns) matrix and is available at the following link: https://docs.google.com/leaf?id=0BxMg4dIPlsq7MzhhMGNjNzMtNGUwYS00NmYzLWI0NDctZjZlNTFiYTEzYWZm&hl=es.

Clusters were generated by randomly allocating the number of genes belonging to each cluster based on Algorithm 2 described in the appendix. A randomly generated profile of treatment effects—scaled by the common within standard deviation of each gene—was added for every gene in the same cluster. The nonscaled profile was generated uniformly between −3 and 3. In this way, differences among treatment means ranged between −3 and 3 times the common within standard deviation for a given gene.

To summarize the effect of the methods to assessing the optimum number of clusters, a linear model was fitted to the difference between the number of clusters in the dataset and its estimation. Hereafter, we will refer to this difference as the *bias*. The factors included in the model were the following: the method used to estimate the number of clusters (*M*), the true number of clusters (*k*), the number of genes (*G*), the number of treatments (*T*), and the number of replicates (*N*). Because each method was applied to the same simulated data, a dataset effect was included in the model as a random effect. Due to the number of terms involved in the adjusted model—main effects and their interaction—the Benjamini-Hochberg algorithm [15] was applied to adjust the raw *P* value in order to control the false discovery rate. The significance level was 0.05. For the significant terms of the model, confidence intervals were calculated for their marginal means. The mixed model was fitted using the *lme* function (*nlme R*-package).

## 4. Results

All the methods compared in this study (except *MClust*) can be applied to the same distance matrix used by the clustering algorithm. Because *gDGC* method uses the Mahalanobis distance to measure the dissimilarity between mean vectors (genes), we decided to base our comparison using this matrix. Mahalanobis distance is a nice metric because it takes into account variances and covariances of attributes. The covariance matrix used to calculate the Mahalanobis distance is the common—pooled—within gene covariance matrix.

Different linkage algorithms are separately analyzed. First we present results for average linkage, then the results for complete linkage. Ward's algorithm was also included in the comparison but results are not shown because of its poor performance. Although *MClust* does not depend on the linkage algorithm, it will appear in the comparison under the subtitles *Average linkage* and *Complete linkage*.

### 4.1. Average Linkage

Table 1 summarizes the ANOVA table for the fitted model when the true number of clusters (*k*) in the dataset is 2 and 10. In both cases, the best model included a variance function to take into account that residual variance was much greater for HOPACH than for the other procedures. The residual standard deviation of *HOPACHm* and *HOPACHc* was around 10 (*k* = 2) to 12 (*k* = 10) times the common standard deviation of the other procedures.

Table 1 shows evidence of differences in the mean bias among methods compared. These differences do not depend on other factors when *k* = 2.  Table 2 summarizes the performance of the methods when *k* = 2. It shows that no matter the input used to the *HOPACH* method (Mahalanobis distances matrix or the mean-GEM), it produces the highest bias, about seven clusters above the true value. On the other hand, only *CCCm*, *CCC*, *gDGC*, and *MClust* had confidence intervals compatible with the unbiasedness hypothesis.

When there is considered the case of moderate number of clusters (*k* = 10), the performance of the methods depends on the number of treatments.  Table 3 shows the mean bias by method, grouped according to the number of treatments. The rank of the methods is almost the same when *T* = 3 or *T* = 5. *HOPACH* overestimated whereas all other methods underestimated the number of clusters. However, as the number of treatments increases (*T* = 5), a differentiation in favour to *gDGC* and *MClust* is apparent.

### 4.2. Complete Linkage

Table 4 summarizes the ANOVA table for the fitted model when the true number of clusters (*k*) in the dataset is 2 and 10. In both cases the best model included a variance function to take into account that residual variance was much greater for *HOPACH* than for the other procedures. The residual standard deviation of *HOPACHm* and *HOPACHc* was around 10 (*k* = 2) to 13 (*k* = 10) times the common standard deviation of the other procedures.

When the true number of clusters in the dataset was 2, the highest interaction terms including *method* were *M* : *G* and *M* : *T*. The mean and 95% confidence interval for the bias for each combination of method and number of genes and of method and number of treatments are shown in Tables 5 and 6, respectively.

As a general remark the increase in *G* is followed by a decrease in the bias. However there are important differences within methods depending on *G*. For *G* = 100, methods which produced estimates compatible with the unbiasedness hypothesis were *CCC*, *CCCm*, *gDGC*, and *MClust*. For *G* = 300, those methods were *HOPACHc*, *CH*, *gDGC*, *Silh*, *CCC*, *CCCm*, and *MClust*.

Considering results shown in Table 6, the increase in the number of treatments is followed by a decrease in bias. As in the previous case there are differences in the performance of the methods depending on *T*. However, no matter *T*, *HOPACH* always overestimated the number of clusters. In the side of best performing methods the list contains *Silh*, *CCC*, *CCCm*, *gDGC*, and *MClust*. When *T* = 5, the previous list is augmented with *CH*.

When the true number of clusters in the dataset was 10, the highest interaction term including method was *M* : *G* : *T*, which logically includes the also significant *M* : *G* and *M* : *T* interaction terms. There is also a second-order significant interaction given by *M* : *N*. The mean and 95% confidence interval for the bias for the combinations of method and number of replicates are shown in Table 7. The corresponding table for combinations of method, number of genes, and number of treatments is shown in Table 8.

As a general remark, when the true number of clusters increases to a moderate number (*k* = 10), all methods underestimated the number of clusters, except *HOPACH*, which consistently overestimated it.

Although a significant interaction was found for the method and the number of replicates, Table 7 shows that there is no change in the ordering of the methods no matter *N*. *Gap*, *H*, *gDGC,* and *MClust* were the lesser negative-biased methods.

To analyze the performance of the methods to estimate the number of clusters regarding the *M* : *G* : *T* interaction term, Table 8 is divided into four blocks defined by the combination levels of *G* and *T*. Within these blocks methods were sorted in descending order of bias. Taking into account the bias in the four blocks there are four methods that always have the lesser bias: *Gap*, *H*, *gDGC*, and *MClust*. Although their order changes in each block, the picture is the same. As in other cases analyzed, *HOPACH* always overestimated, by far, the number of clusters in the dataset.

## 5. Discussion

Two scenarios were considered in this work: when the true number of clusters is very small and when the number is moderate. In the first case (i.e., *k* = 2), some methods estimated the true number of clusters quite well, no matter the linkage algorithm. These methods were *CCC*, *CCCm*, *gDGC*, and *MClust*. Meanwhile, all other methods produced overestimate. Within this group of methods, *HOPACH* (based on the Mahalanobis distance or the average gene-expression matrix) was, by far, the highest biased.

The case when the number of clusters is small is not the most challenging situation because most of clustering methods find the global structure. Moreover, in most microarray experiments the number of clusters will be greater than two. The problem is finding relatively small clusters in the presence of one or more larger clusters [2]. For moderate number of clusters, as could be 10, all methods gave negative-biased estimations of the number of clusters, except *HOPACHm* and *HOPACHc* that were positive biased and very variable. The positive bias of *HOPACH* is consistent with the properties of the median split Silhouette criterion (MSS), which was developed to be more “aggressive" for finding small, homogeneous clusters in large datasets [13].

 Within the negative-biased methods, and according to the simulated scenarios, the results for the average and complete linkage algorithms suggest that the less-biased methods for assessing the number of clusters were *MClust*, *gDGC,* and *Gap*. However, considering all the scenarios there are two methods that consistently appeared in the best groups: *gDGC* and *MClust*.

One disadvantage of *gDGC* compared to *MClust* is that it relays on the availability of replicates. However, in actual microarray applications there are always biological replicates. So, in this context, that limitation is not a problem. Although gDGC is based on the null distribution of the root node of a binary tree, generated by a hierarchical clustering algorithm, and *MClust* is based on the modeling of a multivariate normal mixture, both are theoretically related. Their null model is that there is just one multivariate-normal population. For this reason, both can give, as a result, one cluster. When the null model fails, *MClust* assumes that the dataset is a mixture of samples from several multivariate-normal populations differing in their mean vector and possibly in their covariance matrix, with the number of populations being a parameter to estimate. *gDGC* also assumes that if the dataset is not a sample of a unique multivariate population, then it is a mixture of samples from several multivariate-normal populations. In contrast to *Mclust*, *gDGC* makes the simplified assumption that there is a common covariance matrix as in MANOVA. Nonetheless, an advantage of *gDGC* is that it can drop the assumption of multivariate normality and resample from an empirical estimated null distribution with, of course, additional computational cost.

Another point in favour to *gDGC* is that it is related to a dendrogram, a common way to illustrate relationships among genes (i.e., heatmaps). So, *gDGC* not only estimates the number of groups of genes having the same expression profile but also shows them using the intuitive idea of cutting a dendrogram, making its interpretation straightforward. Because the rule to cut the dendrogram is based of a testing hypothesis framework, it allows the user to calibrate the power of the test selecting the significance level of his/her choice.

*gDGC* and *MClust* are computer intensive methods, and the users will have to face the time cost of their implementations. However, it is possible—for common setups of the number of genes, replicates, number of treatments, and linkage algorithm—to speed up the *gDGC* algorithm having already calculated the appropriate percentile tables of the null distribution of its decision statistic.

In summary, there are not unbiased methods for estimating the number of clusters in a gene-expression matrix within those methods compared in this study. However, within the negative-biased methods, *MClust* and *gDGC* are the best choice.

## Figures and Tables

**Table 1:** Summarized ANOVA table for the terms of the linear model fitted to the bias (estimated minus true number of clusters—*k*—in the gene-expression matrix). Results are shown for *k* = 2 and *k* = 10. Clustering algorithm: average linkage.

Model terms	numDF	denDF	*k* = 2		*k* = 10	
			*F*-value	BH-Adj *P* value	*F*-value	BH-Adj *P* value
*M*	9	720	11.44	<0.0001	159.17	<0.0001
*G*	1	80	12.32	0.0037	21.22	<0.0001
*T*	1	80	3.52	0.1653	64.34	<0.0001
*N*	1	80	0.01	0.9858	3.38	0.1243
*M* : *G*	9	720	2.42	0.0510	2.60	0.0177
*M* : *T*	9	720	1.84	0.1653	10.96	<0.0001
*M* : *N*	9	720	1.71	0.1757	2.21	0.0485
*G* : *T*	1	80	0.01	0.9858	2.40	0.1824
*G* : *N*	1	80	1.62	0.3812	0.01	0.9233
*T* : *N*	1	80	0.49	0.8077	2.57	0.1823
*M* : *G* : *T*	9	720	0.50	0.9858	1.48	0.2050
*M* : *G* : *N*	9	720	0.66	0.9858	1.06	0.4865
*M* : *T* : *N*	9	720	1.79	0.1653	2.09	0.0594
*G* : *T* : *N*	1	80	0.36	0.8268	0.54	0.5333
*M* : *G* : *T* : *N*	9	720	0.25	0.9858	0.79	0.6701

BH-Adj *P* value: refers to the adjusted *P* value according to Benjamini-Hochberg algorithm.

**Table 2:** Estimated mean, standard error, and lower (LB) and upper boundaries (UB) of a 95% confidence interval for the bias for each method applied to the estimation of the number of clusters in the simulated datasets. True number of clusters: *k* = 2. Clustering algorithm: average linkage.

Method	Mean bias	Standard error	LB (95%)	UB (95%)
HOPACHm	7.35	1.07	5.25	9.45
HOPACHc	7.28	1.07	5.18	9.38
CH	0.44	0.10	0.24	0.64
Gap	0.35	0.10	0.15	0.55
Silh	0.34	0.10	0.14	0.54
H	0.22	0.10	0.02	0.42
CCCm	0.16	0.10	−0.04	0.36
CCC	0.16	0.10	−0.04	0.36
gDGC	0.03	0.10	−0.17	0.23
MClust	0.01	0.10	−0.19	0.21

**Table 3:** Estimated mean, standard error, and lower (LB) and upper boundaries (UB) of a 95% confidence interval for the bias for each combination of method (*M*) and number of treatments (*T*). Means of bias are sorted descending within each level of *T*. True number of clusters: *k* = 10. Clustering algorithm: average linkage.

*M*	*T*	Mean bias	Standard error	LB (95%)	UB (95%)
HOPACHm	3	33.00	2.56	32.80	33.20
HOPACHc	3	15.43	2.56	15.23	15.63
MClust	3	−4.68	0.21	−6.78	−2.58
gDGC	3	−4.77	0.21	−6.87	−2.67
Gap	3	−5.00	0.21	−5.20	−4.80
H	3	−5.09	0.21	−5.29	−4.89
CH	3	−6.14	0.21	−6.34	−5.94
Silh	3	−6.84	0.21	−7.04	−6.64
CCC	3	−7.23	0.21	−7.43	−7.03
CCCm	3	−7.25	0.21	−7.45	−7.05
HOPACHm	5	36.82	2.56	34.72	38.92
HOPACHc	5	35.82	2.56	33.72	37.92
gDGC	5	−1.41	0.21	−1.61	−1.21
MClust	5	−2.02	0.21	−2.22	−1.82
Gap	5	−2.84	0.21	−3.04	−2.64
CH	5	−3.11	0.21	−3.31	−2.91
H	5	−3.34	0.21	−3.54	−3.14
Silh	5	−4.07	0.21	−4.27	−3.87
CCCm	5	−6.39	0.21	−6.59	−6.19
CCC	5	−6.39	0.21	−6.59	−6.19

**Table 4:** Summarized ANOVA table for the terms of the linear model fitted to the bias (estimated minus true number of clusters—*k*—in the gene-expression matrix). Results are shown for *k* = 2 and *k* = 10. Clustering algorithm: complete linkage.

Model terms	numDF	denDF	*k* = 2		*k* = 10	
			*F*-value	BH-Adj *P* value	*F*-value	BH-Adj *P* value
*M*	9	720	130.17	<0.0001	265.88	<0.0001
*G*	1	80	4.26	0.0845	11.78	0.0017
*T*	1	80	26.39	<0.0001	57.35	<0.0001
*N*	1	80	0.54	0.5703	28.62	<0.0001
*M* : *G*	9	720	28.29	<0.0001	10.05	<0.0001
*M* : *T*	9	720	19.67	<0.0001	14.43	<0.0001
*M* : *N*	9	720	0.71	0.7031	4.15	0.0001
*G* : *T*	1	80	0.3	0.6331	0.33	0.6046
*G* : *N*	1	80	0.78	0.5063	0.83	0.4169
*T* : *N*	1	80	2.19	0.2289	1.55	0.2897
*M* : *G* : *T*	9	720	2.66	0.0129	3.78	0.0002
*M* : *G* : *N*	9	720	0.82	0.6331	1.27	0.3096
*M* : *T* : *N*	9	720	1.43	0.2508	1.63	0.1631
*G* : *T* : *N*	1	80	2.19	0.2289	2.58	0.1631
*M* : *G* : *T* : *N*	9	720	2.23	0.0419	0.48	0.8888

BH-Adj *P* value: refers to the adjusted *P* value according to Benjamini-Hochberg algorithm.

**Table 5:** Estimated means, standard error, and lower (LB) and upper boundaries (UB) of a 95% confidence interval for the bias for each combination of method (*M*) and number of genes (*G*). The table is sorted in descending order of bias within each level of *G*. True number of clusters: *k* = 2. Clustering algorithm: complete linkage.

*M*	*G*	Mean bias	Standard error	LB (95%)	UB (95%)
HOPACHm	100	14.93	2.08	10.85	19.01
HOPACHc	100	11.14	2.08	7.05	15.22
Gap	100	3.11	0.22	2.69	3.54
H	100	1.93	0.22	1.51	2.35
CH	100	0.68	0.22	0.26	1.10
Silh	100	0.45	0.22	0.03	0.88
CCC	100	0.16	0.22	−0.26	0.58
CCCm	100	0.16	0.22	−0.26	0.58
gDGC	100	0.05	0.22	−0.38	0.47
MClust	100	−0.05	0.22	−0.47	0.38
HOPACHm	300	8.16	2.08	4.08	12.24
H	300	6.41	0.22	5.99	6.83
Gap	300	4.77	0.22	4.35	5.20
HOPACHc	300	3.95	2.08	−0.13	8.04
CH	300	0.11	0.22	−0.31	0.54
gDGC	300	0.09	0.22	−0.33	0.51
Silh	300	0.07	0.22	−0.35	0.49
CCC	300	0.02	0.22	−0.40	0.45
CCCm	300	0.02	0.22	−0.40	0.45
MClust	300	−0.02	0.22	−0.45	0.40

**Table 6:** Estimated mean, standard error, and lower (LB) and upper boundaries (UB) of a 95% confidence interval for the bias of each combination of method (*M*) and number of treatments (*T*). The table is sorted in descending order of bias within each level of *T*. True number of clusters: *k* = 2. Clustering algorithm: complete linkage.

*M*	*T*	Mean bias	Standard error	LB (95%)	UB (95%)
HOPACHm	3	17.25	2.08	13.17	21.33
HOPACHc	3	9.27	2.08	5.19	13.35
H	3	5.84	0.22	5.42	6.26
Gap	3	5.43	0.22	5.01	5.85
CH	3	0.75	0.22	0.33	1.17
Silh	3	0.41	0.22	−0.01	0.83
CCC	3	0.18	0.22	−0.24	0.60
CCCm	3	0.18	0.22	−0.24	0.60
gDGC	3	0.00	0.22	−0.42	0.42
MClust	3	−0.07	0.22	−0.49	0.35
HOPACHm	5	5.84	2.08	1.76	9.92
HOPACHc	5	5.82	2.08	1.74	9.90
H	5	2.50	0.22	2.08	2.92
Gap	5	2.45	0.22	2.03	2.88
gDGC	5	0.14	0.22	−0.29	0.56
Silh	5	0.11	0.22	−0.31	0.54
CH	5	0.05	0.22	−0.38	0.47
CCCm	5	0.00	0.22	−0.42	0.42
MClust	5	0.00	0.22	−0.42	0.42
CCC	5	0.00	0.22	−0.42	0.42

**Table 7:** Estimated mean, standard error, and lower (LB) and upper boundaries (UB) of a 95% confidence interval for the bias of each combination of method (*M*) and number of replicates (*N*). The table is sorted in descending order of bias within each level of *N*. True number of clusters: *k* = 10. Clustering algorithm: complete linkage.

*M*	*N*	Mean bias	Standard error	LB (95%)	UB (95%)
HOPACHm	3	33.00	2.58	27.94	38.06
HOPACHc	3	25.18	2.58	20.12	30.24
Gap	3	−1.95	0.23	−2.40	−1.50
H	3	−2.27	0.23	−2.72	−1.82
gDGC	3	−3.75	0.23	−4.20	−3.30
MClust	3	−3.95	0.23	−4.40	−3.50
CH	3	−5.48	0.23	−5.93	−5.03
Silh	3	−6.57	0.23	−7.02	−6.12
CCCm	3	−7.11	0.23	−7.56	−6.66
CCC	3	−7.23	0.23	−7.68	−6.78
HOPACHm	6	32.39	2.58	27.33	37.45
HOPACHc	6	27.43	2.58	22.37	32.49
Gap	6	−0.89	0.23	−1.34	−0.44
H	6	−1.36	0.23	−1.81	−0.91
gDGC	6	−2.45	0.23	−2.90	−2.00
MClust	6	−2.98	0.23	−3.43	−2.53
CH	6	−3.55	0.23	−4.00	−3.10
Silh	6	−5.25	0.23	−5.70	−4.80
CCCm	6	−7.16	0.23	−7.61	−6.71
CCC	6	−7.23	0.23	−7.68	−6.78

**Table 8:** Estimated mean, standard error, and lower (LB) and upper boundaries (UB) of a 95% confidence interval for the bias of each combination of method (*M*), number of genes (*G*), and number of treatments (*T*). The table is sorted in descending order of bias within each level of *G* and *T*. True number of clusters: *k* = 10. Clustering algorithm: complete linkage.

*M*	*G*	*T*	Mean bias	Standard error	LB (95%)	UB (95%)
HOPACHm	100	3	28.18	3.64	21.05	35.31
HOPACHc	100	3	23.14	3.64	16.01	30.27
Gap	100	3	−2.14	0.32	−2.77	−1.51
H	100	3	−3.50	0.32	−4.13	−2.87
gDGC	100	3	−4.55	0.32	−5.18	−3.92
CH	100	3	−4.91	0.32	−5.54	−4.28
MClust	100	3	−5.09	0.32	−5.72	−4.46
Silh	100	3	−6.82	0.32	−7.45	−6.19
CCCm	100	3	−7.41	0.32	−8.04	−6.78
CCC	100	3	−7.41	0.32	−8.04	−6.78
HOPACHc	100	5	30.05	3.64	22.92	37.18
HOPACHm	100	5	26.95	3.64	19.82	34.08
Gap	100	5	−1.45	0.32	−2.08	−0.82
gDGC	100	5	−2.00	0.32	−2.63	−1.37
MClust	100	5	−2.45	0.32	−3.08	−1.82
H	100	5	−3.05	0.32	−3.68	−2.42
CH	100	5	−3.73	0.32	−4.36	−3.10
Silh	100	5	−4.95	0.32	−5.58	−4.32
CCCm	100	5	−7.09	0.32	−7.72	−6.46
CCC	100	5	−7.36	0.32	−7.99	−6.73
HOPACHm	300	3	40.23	3.64	33.1	47.36
HOPACHc	300	3	16.09	3.64	8.96	23.22
H	300	3	0.36	0.32	−0.27	0.99
Gap	300	3	−1.18	0.32	−1.81	−0.55
gDGC	300	3	−4.14	0.32	−4.77	−3.51
MClust	300	3	−4.23	0.32	−4.86	−3.60
CH	300	3	−6.36	0.32	−6.99	−5.73
Silh	300	3	−7.41	0.32	−8.04	−6.78
CCCm	300	3	−7.45	0.32	−8.08	−6.82
CCC	300	3	−7.45	0.32	−8.08	−6.82
HOPACHc	300	5	35.95	3.64	28.82	43.08
HOPACHm	300	5	35.41	3.64	28.28	42.54
Gap	300	5	−0.91	0.32	−1.54	−0.28
H	300	5	−1.09	0.32	−1.72	−0.46
gDGC	300	5	−1.73	0.32	−2.36	−1.10
MClust	300	5	−2.09	0.32	−2.72	−1.46
CH	300	5	−3.05	0.32	−3.68	−2.42
Silh	300	5	−4.45	0.32	−5.08	−3.82
CCCm	300	5	−6.59	0.32	−7.22	−5.96
CCC	300	5	−6.68	0.32	−7.31	−6.05

## Appendix

Algorithm 1 A1 For the generation of a simulated gene-expression matrix (sGEM), one has the following.

1. Initialize groups = number of treatments, genes = number of genes, replicates = number of replicates, and set index *g*, *r*, and *i* to 0.
2. Let *i* = *i* + 1.
3. Randomly choose the index *k* that points to a row of the rGEM (residual matrix of gene expressions).
4. Initialize *j* = 0.
5. Let *g* = *g* + 1.
6. Let *r* = *r* + 1.
7. Randomly choose the index m that points to a column of rGEM.
8. Set *j* = *j* + 1 that points to the columns of the sGEM (simulated matrix of gene-expression).
9. Let sGEM[*i*, *j*] = rGEM[*k*, *m*] × *s*[*k*] + *m*[*k*, 1] + *E*[*i*, *g*] × *s*[*k*] (*E* is the matrix of treatment effects).
10. If *r* < replicates, go to 7; else if *g* < groups, go to 6; else if *i* < genes, go to 3; else end.

Algorithm 2 A2 Genes belonging to each cluster will be generated by randomly assigning a row from the rGEM according to the following algorithm.

1. Let *k* be the number of clusters to construct, *N* the total number of candidate genes, and *L* a list of integers of size *N* indexing the genes.
2. Initialize *L* with the sequence 1 … *N*.
3. “Order at random” *L*.
4. Select a set *h* of (*k* − 1) indexes between 1 and *N*. If some of this (*k* − 1), are equal find another set of indexes.
5. Sort *h*, in ascending order.
6. *L*[1] is the index of first and *L*[*h*[1] − 1] the index of the last gene belonging to cluster 1, and so on until *L*[*h*[*k* − 1] is the index of first and *L*[*N*] the index of last gene belonging to cluster *k*.
